# Urinary microRNAs as non-invasive biomarkers for toxic acute kidney injury in humans

**DOI:** 10.1038/s41598-021-87918-0

**Published:** 2021-04-28

**Authors:** Fathima Shihana, Wilson K. M. Wong, Mugdha V. Joglekar, Fahim Mohamed, Indika B. Gawarammana, Geoffrey K. Isbister, Anandwardhan A. Hardikar, Devanshi Seth, Nicholas A. Buckley

**Affiliations:** 1 Clinical Pharmacology and Toxicology Research Group, Biomedical Informatics and Digital Health, Faculty of Medicine and Health, The University of Sydney, Sydney, NSW Australia; 2 South Asian Clinical Toxicology Research Collaboration, Faculty of Medicine, University of Peradeniya, Peradeniya, Sri Lanka; 3 Centenary Institute of Cancer Medicine and Cell Biology, The University of Sydney, Sydney, NSW Australia; 4 Diabetes and Islet Biology Group, School of Medicine, Western Sydney University, Campbelltown, NSW Australia; 5 Allied Health Sciences, Department of Pharmacy, University of Peradeniya, Peradeniya, Sri Lanka; 6 Australian Kidney Biomarker Reference Laboratory, Department of Nephrology, Prince of Wales Hospital and Clinical School, University of New South Wales, Sydney, Australia; 7 Clinical Toxicology Research Group, University of Newcastle, Newcastle, NSW Australia; 8 Department of Science and Environment, Roskilde University, Roskilde, Denmark; 9 Discipline of Clinical Medicine and Addiction Medicine, Faculty of Medicine and Health, The University of Sydney, Sydney, NSW Australia; 10 Drug Health Services, Royal Prince Alfred Hospital, Sydney, NSW Australia

**Keywords:** miRNAs, Acute kidney injury, Diagnostic markers, Toxicology

## Abstract

MicroRNAs in biofluids are potential biomarkers for detecting kidney and other organ injuries. We profiled microRNAs in urine samples from patients with Russell’s viper envenoming or acute self-poisoning following paraquat, glyphosate, or oxalic acid [with and without acute kidney injury (AKI)] and on healthy controls. Discovery analysis profiled for 754 microRNAs using TaqMan OpenArray qPCR with three patients per group (12 samples in each toxic agent). From these, 53 microRNAs were selected and validated in a larger cohort of patients (Russell’s viper envenoming = 53, paraquat = 51, glyphosate = 51, oxalic acid = 40) and 27 healthy controls. Urinary microRNAs had significantly higher expression in patients poisoned/envenomed by different nephrotoxic agents in both discovery and validation cohorts. Seven microRNAs discriminated severe AKI patients from no AKI for all four nephrotoxic agents. Four microRNAs (miR-30a-3p, miR-30a-5p, miR-92a, and miR-204) had > 17 fold change (*p* < 0.0001) and receiver operator characteristics area-under-curve (ROC-AUC) > 0.72. Pathway analysis of target mRNAs of these differentially expressed microRNAs showed association with the regulation of different nephrotoxic signaling pathways. In conclusion, human urinary microRNAs could identify toxic AKI early after acute injury. These urinary microRNAs have potential clinical application as early non-invasive diagnostic AKI biomarkers.

## Main

MicroRNAs are involved in all cellular functions, including development, differentiation, growth, metabolism and regulate numerous physiological and pathophysiological processes^1,2^. MicroRNAs are endogenous, non-coding RNAs, 21–25 nucleotides in size that regulate gene expression by binding to target messenger RNAs (mRNAs). Cells secrete microRNAs into the extracellular environment to function as regulators, carrying genetic information from one cell to another, or following cell injury^3^. These extracellular microRNAs are potential biomarkers for a variety of diseases including acute kidney injury (AKI)^4^ and can also be mediators of disease.

Acute kidney injury is associated with changes in the expression of specific microRNAs. While some microRNAs contribute to the pathogenesis and progression of AKI, others may serve as protective molecules^5^. As signalling molecules, microRNAs mediate cell–cell communications, and form a complex regulatory network^2,6^. They play a critical role as anti-apoptotic, pro-apoptotic and inflammatory molecules to facilitate the development of renal injury in nephrotoxic AKI^7,8^. Thus, microRNAs have not only emerged as novel biomarkers for AKI, but they also hold promise as potential therapeutic targets.Various microRNAs regulate different nephrotoxic pathways, for example NF-κB dependent inflammatory responses, TGF-β signalling, and oxidative stress^9–11^. Thus, different mechanisms of nephrotoxicity including renal tubular toxicity, inflammation, glomerular damage, crystal nephropathy, and thrombotic microangiopathy from different toxic agents would be expected to lead to variable microRNA expression.

Traditional biomarkers of AKI such as serum creatinine (SCr) or urine output are both late indicators of kidney injury^12,13^. Further, the rapid rise of SCr after oxalic acid, paraquat, and glyphosate surfactant herbicide (glyphosate) poisoning suggests other mechanisms occur beyond loss of renal function^14–16^. Protein biomarkers which reflect the underlying renal glomerular and/or tubular damage during nephrotoxicity such as β2-microglobulin, cystatin C (Cys C), albumin, neutrophil gelatinase-associated lipocalin (NGAL), kidney injury molecule-1 (KIM-1), interleukin-18 (IL-18) have highly variable diagnostic performance in these three poisonings^14–16^. New biomarkers that are consistent for early prediction and/or detection of diverse causes of nephrotoxic AKI are needed, whilst those specific to particular toxins may provide further understanding of the mechanisms of nephrotoxicity.

Acute kidney injury is a common clinical complication following envenoming and poisoning^14,16–18^. Our main aim was to explore if urinary microRNAs can be used as early renal biomarkers following nephrotoxic-AKI (snakebite envenoming, oxalic acid, paraquat, and glyphosate poisoning). We also investigated whether microRNA signatures identify some common mechanisms of nephrotoxicity using target mRNAs and pathway analysis.

## Results

### Global profiling of urinary microRNAs detects AKI after different causes of poisoning

**Figure 1:**
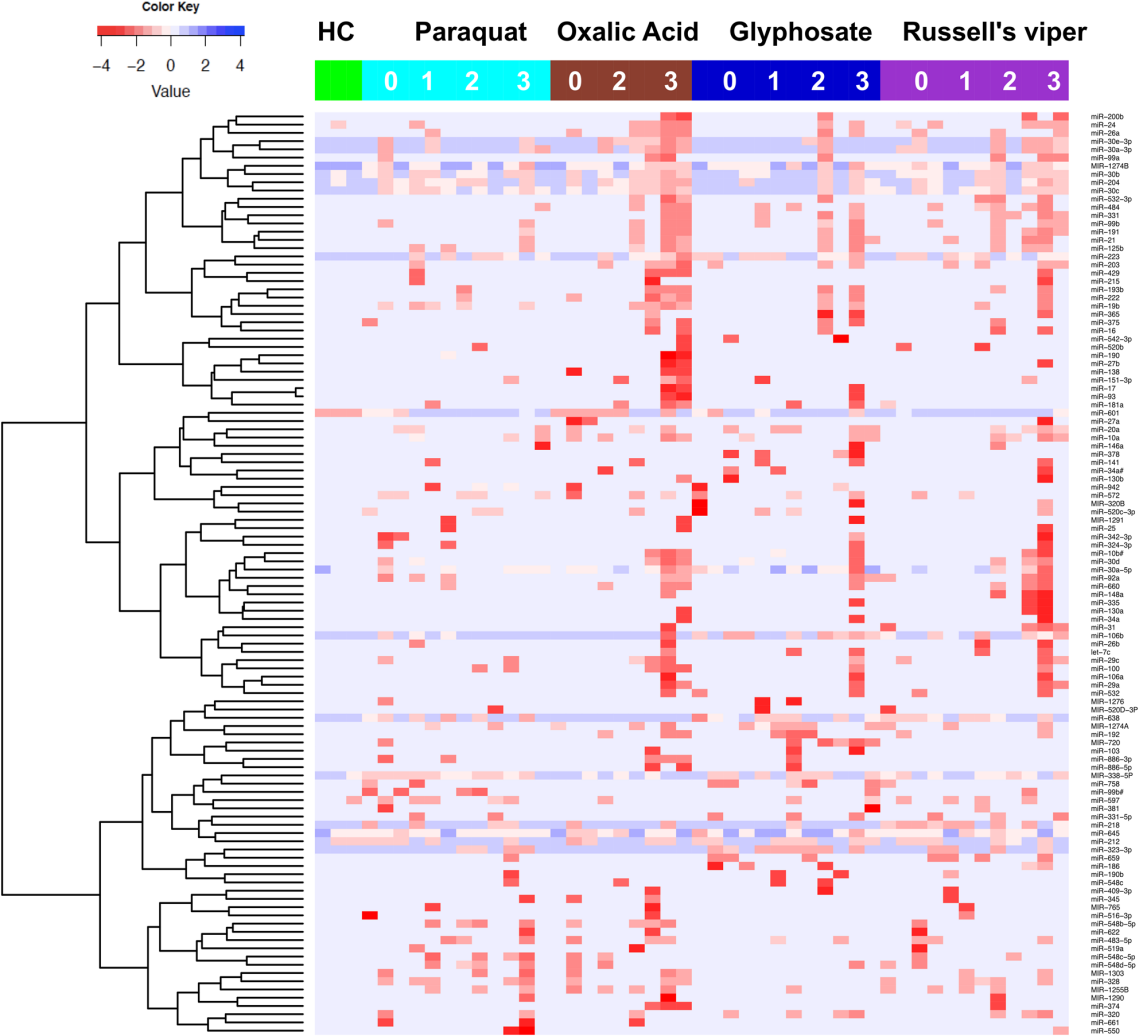
Urinary microRNAs detect AKI after different causes of poisoning. Supervised hierarchical clustering heat map of differentially expressed microRNAs (n=112) distinguished patients with severe AKI from NOAKI and healthy controls. Green, cyan, maroon, dark blue and purple indicates healthy controls, paraquat, Glyphosate, Oxalic acid and Russell’s viper bite respectively. AKI stages are numbered as 0, 1, 2 and 3 for NOAKI, AKIN1, AKIN2 and AKIN3 respectivly. Rows represent microRNAs (normalised Ct) and columns represent the individual samples from healthy controls and patients.

We identified 112 differentially expressed urinary microRNAs from the discovery cohort of 48 patients with Russell’s viper envenoming or acute self-poisoning following paraquat, glyphosate or oxalic acid. The cohort included those who developed no injury (NOAKI), mild injury (AKIN1), moderate injury (AKIN2) or severe injury (AKIN3) (n = 3 per group and n = 12 per each toxins) as well as healthy controls (n = 3). The expression of microRNAs was significantly upregulated in urine samples of patients who developed moderate to severe injury (AKIN2/3) compared to the NOAKI and healthy controls in the discovery cohort. Supervised hierarchical clustering heat map demonstrates the differentially expressed microRNAs between patients with moderate to severe AKI (AKIN2/3) from NOAKI and healthy controls (Fig. 1). Based on our discovery analysis or from past literature, we selected a set of 53 microRNAs (> 4 fold change and *p* < 0.01; Supplementary Table 1) for further validation in independent cohorts for each toxins (Russell’s viper bite = 53, paraquat = 51, glyphosate = 51, oxalic acid = 40, and 27 healthy controls) (see “Methods” section and Supplementary Table 2 for details).

### Clustering of microRNA in severe cases from different causes of AKI in the validation cohort

**Figure 2:**
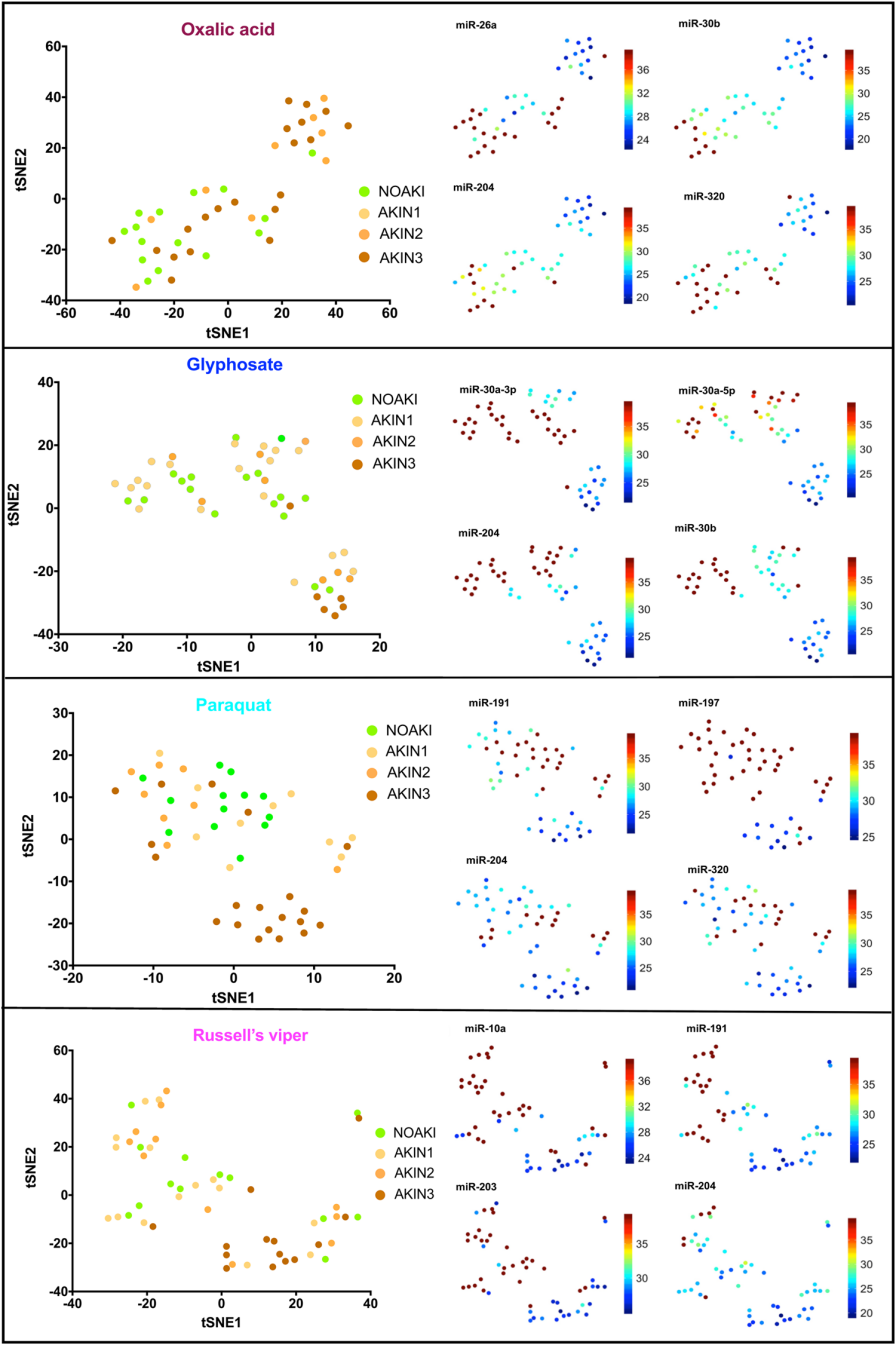
tSNE plots show the clusters of individual microRNAs with NOAKI, AKIN1, AKIN2 and AKIN3 caused by different poisons. Left panels show plots of the non-linear tSNE map of samples in validation cohort using each specific set of differentially expressed microRNAs for different poisons (oxalic acid, glyphosate, paraquat) and Russell’s viper bite, (Supplementary Table 3). Plots were generated using 4-dimensional principal component space into two dimensions showing tSNE analysis. Each point represents a single sample. The right panel shows the expression of the top four most significantly altered microRNAs for each poison presented as individual tSNE-projections. Blue to red presented in the color bar indicate high to low microRNA expression (data shown in normalized Ct values, therefore a lower value represents a higher expression) respectively.

Forty four microRNAs were significantly upregulated in AKI in one or more nephrotoxic agents (oxalic acid = 25, glyphosate = 10, paraquat = 34 and Russell’s viper = 25) compared to NOAKI. Thirty two microRNAs were upregulated for two or more agents (Supplementary Table 3). Different sets of microRNAs for different toxins distinguished patients with AKI from NOAKI in the validation cohort (Fig. 2). Twelve microRNAs distinguished AKI from NOAKI for only one specific toxin [oxalic acid (n = 2), paraquat poisoning (n = 5), Russell’s viper envenoming (n = 5)] (Supplementary Table 3).

### Urinary microRNAs provide signals distinguishing both kidney injury severity and toxic agents

**Figure 3:**
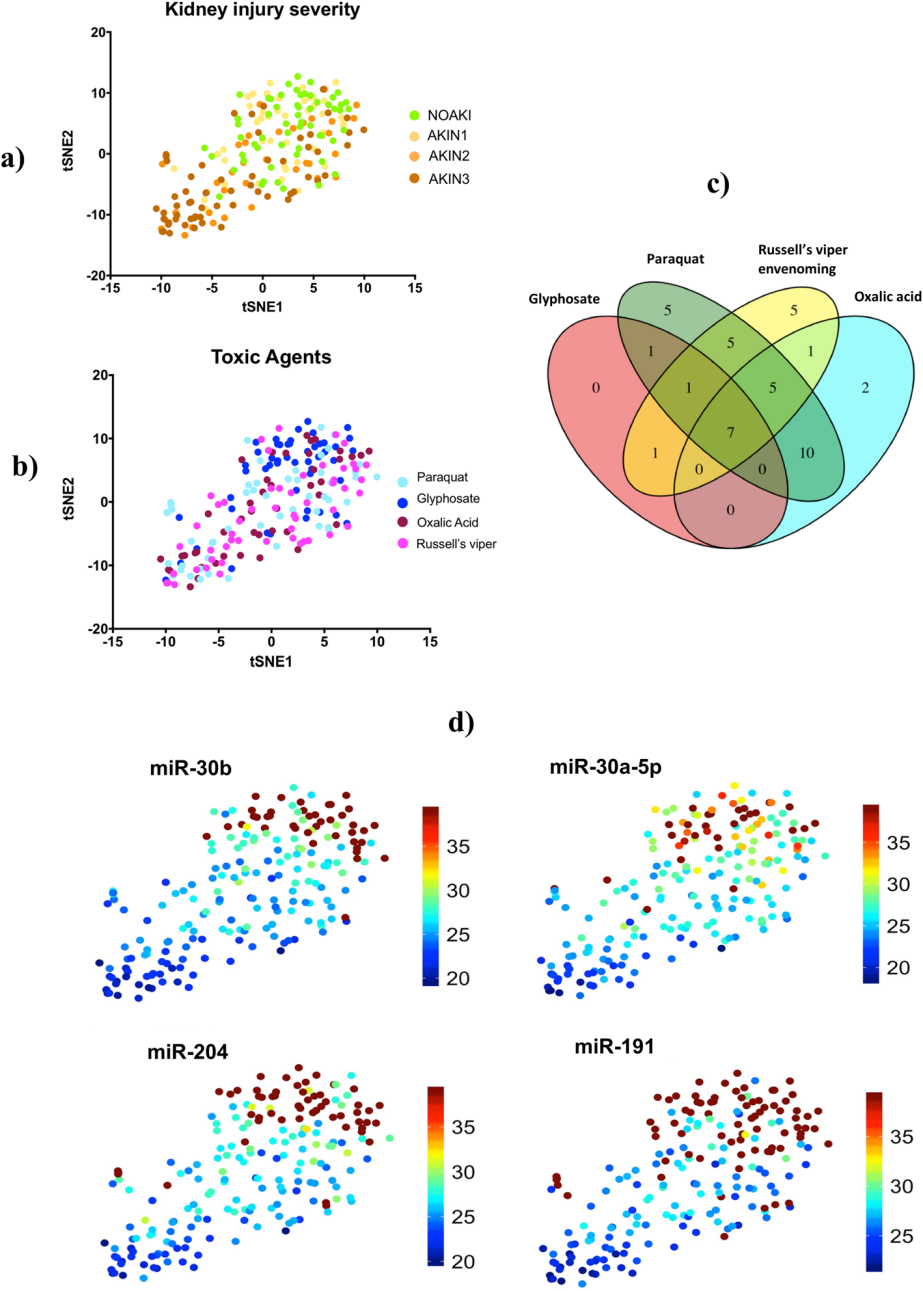
Plot of the non-linear tSNE map of samples from 4-dimensional principal component space into two dimensions showing tSNE analysis applied using all (n=43) differentially expressed microRNAs (Supplementary Table 3). (**a**) tSNE plots show the clusters of all different microRNAs in different AKI stages: NOAKI, AKIN1, AKIN2 and AKIN3 caused by all poisons. (**b**) tSNE plots show the clusters for different poisons: oxalic acid, glyphosate, paraquat and Russell’s viper envenoming in validation cohort. (**c**) Venn Diagram demonstrating overlap of 43 altered microRNAs in AKI (> 2.8 fold change, *p* < 0.05) amongst the nephrotoxic agents. microRNAs altered in different agents: Oxalic acid = 25, glyphosate = 10, paraquat = 34 and Russell’s viper = 25. (**d**) tSNE plots show best four microRNAs clusters in AKIN2/3 patients in all four nephrotoxic agents. Blue to red presented in the color bar indicates high to low microRNA expression (data shown in normalized Ct values, therefore a lower value represents a higher expression) respectively.

**Figure 4:**
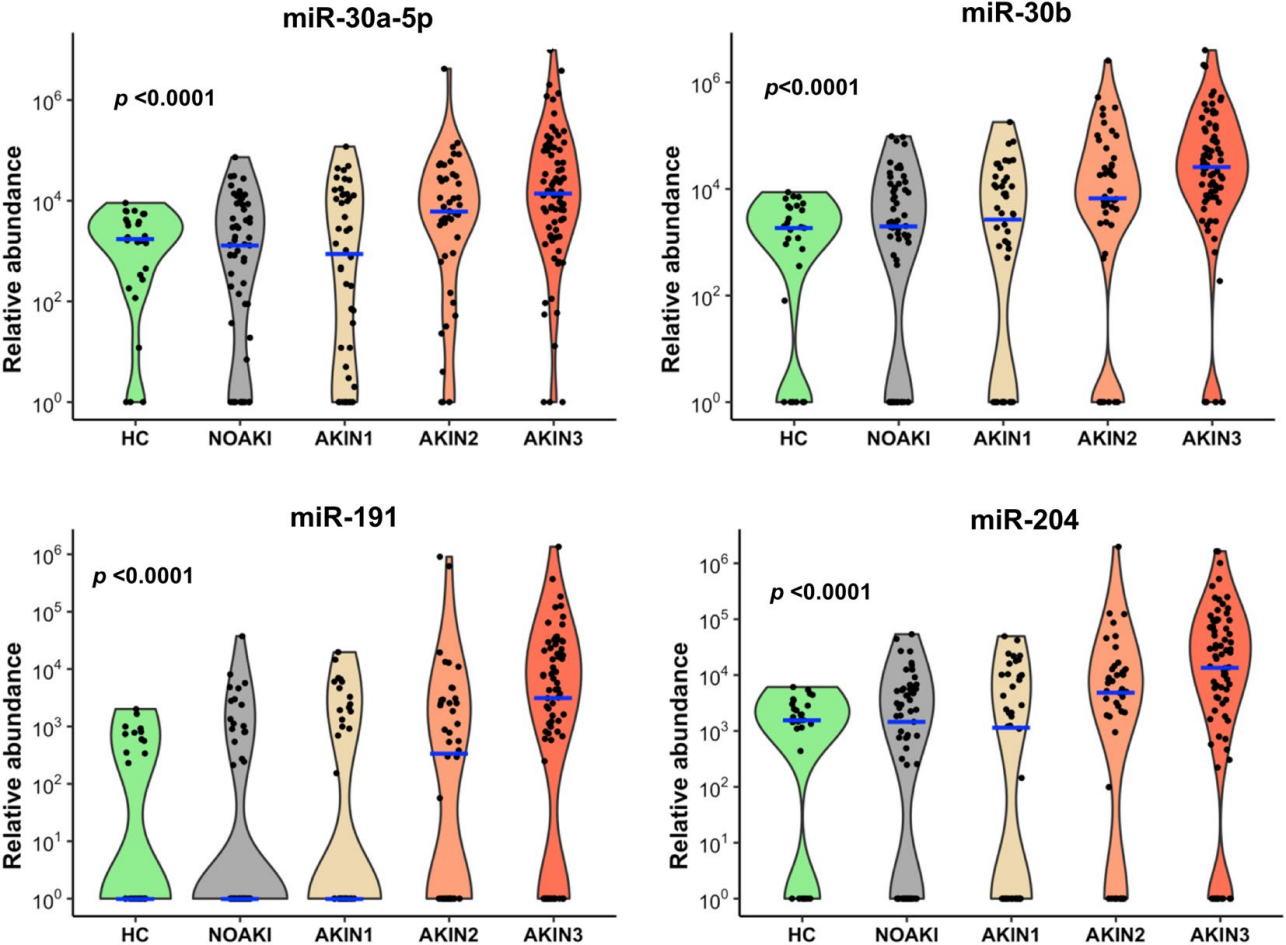
Top four microRNAs associated with AKI for all toxic agents. Violin plots show these microRNAs having increased abundance with increasing AKI severity in all four toxins. Blue lines indicate the medians and each black dot presents a different sample from validation cohort. The *y*-axis is relative abundance from the detectable limit (2^(39-Ct)^, Ct of 39 is the threshold of detectability). MicroRNA expressions were normalised using two spike control ath-microRNAs (*ath*-miR-159a and *ath*-miR-172a). *p* is the alternative *p* value from Jonckheere–Terpstra nonparametric ordered test.

Differentially expressed microRNAs from all four nephrotoxic agents were examined together to visualize separation of patients with AKI from NOAKI using tSNE clustering analysis (Fig. 3a). Despite the selection of differentially expressed microRNAs based on comparisons across all causes of kidney injury for validation, there was still apparent glyphosate clustering in the tSNE plot (Fig. 3b). Seven common microRNAs discriminated AKIN2/3 patients from NOAKI for all four nephrotoxic agents (Fig. 3c, Supplementary Table 3). Of these seven microRNAs, four (miR-30a-5p, miR-30b, miR-191, and miR-204, Fig. 3d) had a greater than 17-fold change (*p* < 0.0001) and ROC-AUC = 0.72 (Table 1). Logistic regression combination of four and all seven microRNAs in NOAKI versus AKIN2/3 patients showed AUC-ROC values of 0.75 and 0.77 respectively (Supplementary Figure 1). The four microRNAs showed an association with increasing disease severity in all poisoning (Jonckheere–Terpstra nonparametric ordered test, *p* < 0.0001, Fig. 4). A schematic diagram shows the initial discovery, through validation and selection of four common microRNAs in all four toxic agents (Supplementary Figure 2).

**Table 1:** Diagnostic performance of the seven microRNAs in all causes of AKI (NOAKI vs. AKIN2/3).

miR	Fold change (2^^ΔCt^)	*p* value	AUC (95% CI)
miR-30a-5p	19.7	< 0.00001	0.72 (0.66–0.79)
miR-30b	17.1	< 0.00001	0.72 (0.66–0.79)
miR-191	32.0	< 0.00001	0.72 (0.66–0.78)
miR-204	17.1	< 0.00001	0.72 (0.65–0.78)
miR-30a-3p	22.6	< 0.00001	0.70 (0.64–0.77)
miR-660	8.0	< 0.00001	0.64 (0.59 0.69)
miR-423-5p	5.7	< 0.00001	0.60 (0.56–0.64)

Diagnostic performance of the seven microRNAs that distinguished AKI in all poisoning was assessed using normalized Ct values. *p* values represent Mann–Whitney-U between NOAKI (n = 57) versus AKIN2/3 (n = 94).

### Identification of target genes and pathway analysis

**Figure 5:**
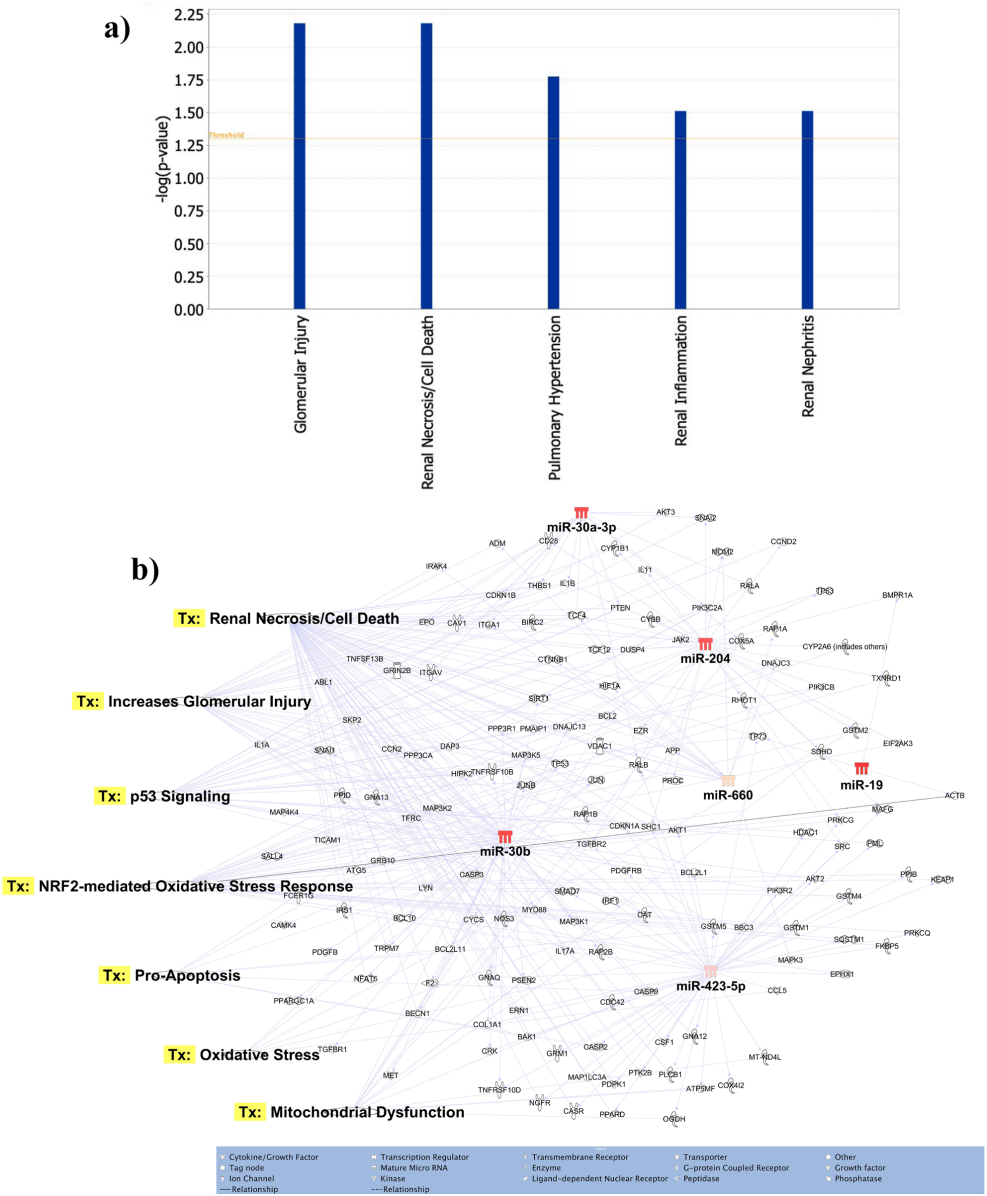
Functions and nephrotoxic pathways associated with target mRNAs of seven differentially expressed microRNAs in toxic-AKI. We analyzed the expression of the seven microRNAs using co-analysis in Ingenuity Pathway Analysis (IPA), (QIAGEN, Inc., https:// targetexplorer.ingenuity.com/) and a) shows the main disease and functions of the differentially expressed microRNAs. We used IPA to identify target mRNA and microRNA-mRNA interactions. Target mRNAs were overlaid on ‘Toxic’ functions derived from select microRNAs using ‘Pathway design’ option in IPA. The top seven toxic function pathway (Tx) networks identified for microRNA-mRNA interactions are presented here.

The seven most differentially expressed microRNAs target glomerular injury, renal cell death/necrosis, renal inflammation and renal nephritis as major disease/functions (Fig. 5a). For the seven differentially expressed microRNAs a total of 586 potential target mRNAs specific to kidney disease/functions were identified via Ingenuity Pathway Analysis (data not shown). These differentially expressed microRNAs targeted many genes associated with the regulation of different nephrotoxic signalling pathways (Fig. 5b). The key pathways targeted by these microRNAs were oxidative stress, renal cell death/necrosis, oxidative stress, apoptosis and mitochondrial dysfunction (Fig. 5b).

## Discussion

We report urinary microRNA signatures for nephrotoxicity in patients with four very different toxic agents (snake envenoming, pesticide and chemical poisoning) in humans. We identified seven common microRNAs that correlated with kidney injury across all four nephrotoxic agents. Another 36 microRNAs provided signals for certain nephrotoxic causes, but not for all agents. We found many of the target mRNAs of these seven common microRNAs are involved in cellular pathways that promote inflammation, apoptosis and oxidative stress.

Very few studies report on multiple agents in nephrotoxic AKI. A recent report on drug induced-AKI in rats using three very different nephrotoxic compounds, cisplatin (proximal tubule) puromycin (glomerulus) and N-phenylanthranylic acid (collecting ducts), identified microRNAs specific to each agent, but did not find any commonly expressed microRNAs across all three^19^. Other studies reported on single agents, for example, cisplatin-induced AKI in rats showed higher expression of urinary miR-191 and miR-30a-5p^20^, and urinary levels of miR-423 were significantly higher in both paracetamol overdose patients with AKI compared with healthy controls and in paracetamol overdose patients without AKI^21^.

We also noted some urinary microRNAs we identified are also observed in non-toxic AKI and other kidney diseases as AKI biomarkers. Urinary miR-423 expression increased in critically ill patients (intensive care unit-AKI)^22^. Patients with glomerular disease such as idiopathic nephrotic syndrome and focal segmental glomerulosclerosis had high levels of miR-30a-5p in urine compared to controls^23–25^. MiR-204 was differentially expressed in urine of patients with chronic acute cellular rejection, characterized by interstitial fibrosis and tubular atrophy, compared with patients with normal histology and functioning allografts^26^.

We studied four very different toxins covering a broad range of mechanisms including ischemia–reperfusion (Russell’s viper bite), uncoupling of oxidative phosphorylation (glyphosate), free radical generation (paraquat), and crystal formation (oxalic acid). However, all these poisonings or envenoming cause mitochondrial damage and inflammation triggering oxidative stress^27^. Oxidative stress, both directly and indirectly affects all facets of the kidney, including vascular reactivity and renal hemodynamics, glomerular filtration and tubular reabsorption and secretion in all nephron segments^28^. During poisoning, oxidative stress signalling alters all these processes and promotes damage pathways that lead to cellular apoptosis, necrosis, altered gene expression, progression of tissue damage, promotion of fibrosis and abnormal kidney function^27,28^. We identified several target mRNAs of the altered microRNAs were associated with mitochondrial dysfunction, apoptosis, oxidative stress and necrosis/cell death.

Most nephrotoxic agents induce proximal tubular injury. The increased concentration of microRNAs in urine may be due to leakage of microRNAs from proximal tubular cells with corresponding decreased levels of microRNAs in the injured tissue^20^, but we did not test this due to unavailability of kidney tissue. Other studies in cisplatin and gentamicin-induced proximal tubular injury showed increased miR-191 and miR-30 family in urine with corresponding decreased expression in tissue^20,29,30^. In human miR-30a-3p, miR-30b, miR-30c, miR-30e-3p had lower expression in acute kidney rejection biopsies compared to normal allograft biopsies^31^.

Interestingly, there are no studies highlighting urinary miR-204 in AKI, despite its renal specific expression^32,33^. MiR-204, that we found common across the four nephrotoxic agents, is involved in the regulation of epithelial-mesenchymal transition by targeting specificity protein 1 (SP1) in the tubular epithelial cells after ischemia–reperfusion injury^34^, and controls local inflammation by regulating interleukin-6 (IL-6) receptor expression^35^. MiR-204 protects interstitial tissue of renal tubules from chronic fibrotic change^34^.

One of our study limitations is that we had a relatively small sample size in the discovery cohort, but we validated the most differentially expressed microRNAs obtained from the discovery results in a much larger cohort. Furthermore, rather than only measuring previously studied microRNAs, we globally profiled 754 microRNAs without ‘a priori’ approach and hence successfully discovered novel microRNAs associated with nephrotoxic agents-induced AKI. We were unable to measure microRNA expression in kidney tissue as biopsies were not done on our patients limiting the study to some extent. As for most studies in patients, we did not have baseline serum creatinine measurement prior to injury and had to use the lowest level measured at any time which may underestimate the degree of injury.

The major strengths of this study are the comprehensive microRNA analysis of kidney injury with four different toxins, global profiling of microRNAs and validation in independent sets of samples using the same analysis platform. Use of multi-centre prospective study samples from five different geographic regions of Sri Lanka is also a strength of the study for generalisability of results. Another strength of the study is that most patients’ samples were taken within 8 h of the injury, a timeframe important for clinical prediction of AKI and guiding treatment plans. Therefore, microRNAs identified in our study can readily serve as potential biomarkers for AKI.

In conclusion, urinary microRNA profiling shows promise in identifying nephrotoxicity after severe AKI in humans. Signature microRNAs including miR-30a-5p, miR-30b, miR-191 and miR-204, could be promising early biomarkers for the detection of toxic AKI. Urinary microRNAs have potential clinical applications as early non-invasive biomarkers for AKI, toxic-AKI, and also for snake envenoming.

## Methods

We included patients with Russell’s viper envenoming or who ingested oxalic acid, paraquat, or glyphosate from a multicenter cohort study in Sri Lanka^16,17,36^. These patients presented to general hospitals between October 2010 and February 2014. Participants completed a written informed consent prior to inclusion in this study. We included patients who provided admission urine samples (collected within 8 h of ingestion). Details of sample collection and storage are described^16,17,36^. AKI was defined by serum creatinine (SCr) and categorized based on Acute Kidney Injury Network (AKIN) criteria^37^. In brief, definition of AKIN stage 1 (mild): an absolute increase in SCr of more than or equal to 0.3 mg/dl or a relative increase by more than or equal to 150 to 200% (1.5 to 2 fold) from baseline, AKIN stage 2 (moderate): a relative increase in SCr of > 200 to 300% (> 2 to 3 fold) from baseline and AKIN stage 3 (severe): a relative increase in SCr of > 300% (> 3 fold) from baseline (or an absolute increase of SCr by more than or equal to 4.0 mg/dl). We used baseline SCr as the lowest level measured at any time (hospital stay or follow-up) because information on pre-exposure was not available.

This study was approved by the Human Research Ethics Committees of the University of New South Wales (Sydney), Australia and the Faculty of Medicine, University of Peradeniya, Sri Lanka. We confirm that all methods were performed in accordance with the relevant guidelines and regulations.

### Sample selection for global profiling and independent validation phase

We used urine samples from patients with acute self-poisoning following paraquat, oxalic acid and glyphosate, and patients who were envenomed by Russell’s vipers. We grouped patients into those who developed no kidney injury (NOAKI), mild injury (AKIN1) moderate injury (AKIN2) or severe injury (AKIN3) (n = 3 per group, n = 12 per each toxin) as well as healthy controls (n = 3) and profiled for microRNAs using the TaqMan OpenArray quantitative real-time PCR (qPCR) platform. We selected a panel of 53 microRNAs (46 significantly different microRNAs between AKI and NOAKI in all toxins in discovery cohort with fold change > 4.0, *p* < 0.01; seven from literature and two were stage-specific spike-in controls and one *A. thaliana* (ath-miR-394a) negative microRNA control) (Supplementary Table 1). These were validated in a larger independent cohort of patients (Russell’s viper bite = 53, paraquat = 51, glyphosate = 51, oxalic acid = 40) and 27 healthy controls (Supplementary Table 2).

### RNA extraction

Total RNA (including small RNA species) was extracted from 200 µl urine by first mixing with 500 µl of TRIzol (ThermoFisher Scientific, USA) and 100 µl chloroform (Sigma Aldrich, Hamburg, Germany), followed by using the RNeasy-HT Kit (Qiagen, Hilden, Germany) as per the manufacturer’s instructions on a QiaCube-HT robotic RNA isolation system. Samples were also spiked in with 2.5 µl of 50 nM synthetic control microRNA *ath*-miR-172a (Sigma Aldrich, Hamburg, Germany) during RNA extraction procedure. The quantity of total RNA was measured using the NanoDrop spectrophotometer.

### OpenArray panels—quantitative real-time PCR

Complementary (c)DNA was synthesised from total RNA using Megaplex RT Primers, Human Pool A and Pool B (ThermoFisher Scientific, USA) and reagents from the TaqMan microRNA Reverse Transcription Kit (ThermoFisher Scientific, USA). Each reverse transcription (RT) reaction contained a final volume of 7.5 μl (100 ng total RNA input). Pre-amplification was performed using Megaplex PreAmp Primers, Human Pool A and B (ThermoFisher Scientific, USA) and TaqMan PreAmp Master mix (ThermoFisher Scientific, USA). Pre-amplified product was diluted 1:40 in 0.1X TE buffer (pH 8.0). Diluted product was then combined with TaqMan OpenArray PCR master mix at a 1:1 ratio and loaded onto TaqMan OpenArray Human microRNA Panel (ThermoFisher Scientific, USA) using the AccuFill system. RT-qPCR was completed using the QuantStudio 12 K Flex System (Life Technologies, Foster City, CA, USA)^38^. Validation PCR was carried out using the Custom TaqMan OpenArray (ThermoFisher Scientific, USA) according to the manufacturer’s ‘low-sample-input’ protocol with custom microRNA RT primer pools, RT spike-in microRNA (*ath*-miR-159a), custom PreAmp Primer Pools and PCR master mix (supplied with the custom OpenArray panel).

### Data analysis

High throughput data generated from TaqMan OpenArray RT-qPCR data were uploaded into ThermoFisher Connect for global normalisation and to set a threshold point. MicroRNAs that were undetectable or had an Amp Score < 1.24 and Cq confidence < 0.6 were eliminated, as per the pre-defined QC criteria for this study. A cut-off threshold cycle (Ct) value of 39 was defined. Validation panel data were directly imported into Excel and normalised using the two spike controls (*ath*-miR-159a and *ath*-miR-172a).

### Statistical analysis

The relative cycle change (ΔCt = Ct_Healthy Control_ − Ct_Patient Sample_) between each group was calculated using normalised Ct-values and the statistical significance (*p* value) was calculated by Student t-tests in Excel. We used semi-supervised (samples-supervised and microRNA expression-unsupervised) cluster heatmaps to show the microRNA expression in different toxic agents by grouping the samples as toxic agents with the severity of AKI stages (supervised). That allows clustering of microRNAs according to expression in each sample. MicroRNAs showing statistically significant changes over ± 4 fold at *p* < 0.01 were selected and measured on the custom OpenArray panel. R statistical program was used to plot heatmaps and t-distributed Stochastic Neighbour Embedding (tSNE) plots to visualise differentially expressed microRNAs, through using R package Heatplus and Rtsne respectively, along with other supportive packages for graphics and resampling. Area under the receiver operator characteristics curve (AUC-ROC) analysis was calculated using GraphPad Prism 8 to evaluate the diagnostic value of candidate microRNAs. Logistic AUC-ROC was calculated to evaluate the diagnostic performance of the seven microRNAs using R statistical program (Proc package). Relative abundance was calculated from the detectable limit (2^^(39-Ct)^). The Jonckheere-Terpstra non-parametric ordered test (with ordering of the populations as follows: Healthy controls, NOAKI, AKIN1, AKIN2, AKIN3) was performed to identify statistically significant trend between independent groups.

### Pathway analysis

We analyzed the expression of the seven microRNAs using co-analysis in Ingenuity Pathway Analysis (IPA), (QIAGEN, Inc., https://targetexplorer.ingenuity.com/) to find the main disease association and functions of these differentially expressed microRNAs. Target mRNAs of these differentially expressed microRNAs were examined using microRNA target filter option in IPA (filter criteria: Renal and urological disease, all molecular types, Pathways: transport, apoptosis, cellular growth, development and proliferation, cellular stress and injury, ingenuity toxicity list). The target mRNAs were then overlaid on ‘Disease and function’ and examined for significant functions and involvement in signalling pathways using Tox function in pathway analysis. Finally, the top pathway and the targets were overlayed with the significantly altered microRNAs to plot the network diagram.

## Supplementary information

Supplementary Informations.

## Data Availability

The datasets generated during and/or analysed during the current study are available from the corresponding author on reasonable request.
