# Supplementary material for: Urinary microRNAs as non-invasive biomarkers for toxic acute kidney injury in humans
Source: Sci Rep. 2021 Apr 28;11:9165. doi: 10.1038/s41598-021-87918-0 (PMC8080685; doi:10.1038/s41598-021-87918-0)
Supplement: Supplementary file 1 — Supplementary Informations. [file 41598_2021_87918_MOESM1_ESM.docx]

**Urinary microRNAs as non-invasive biomarkers for toxic acute kidney injury in humans**

Fathima Shihana*^1,2,3^, Wilson K.M.Wong^4^, Mugdha V. Joglekar^4^, Fahim Mohamed^1,2,5^, Indika B. Gawarammana^2^, Geoffrey K. Isbister^6^, Anandwardhan A. Hardikar^ⱡ4^, Devanshi Seth^ⱡ3,7,8^, Nicholas A. Buckley*^ⱡ1,2^

^1^The University of Sydney, Discipline of Pharmacology, Sydney Medical School, NSW, Australia

^2^University of Peradeniya, South Asian Clinical Toxicology of Research Collaboration, Faculty of Medicine, Sri Lanka

^3^The University of Sydney, Centenary Institute of Cancer Medicine & Cell Biology, NSW, Australia

^4^Diabetes & Islet Biology Group, School of Medicine, Western Sydney University, Campbelltown, New South Wales, Australia

^5^University of Peradeniya, Allied Health Sciences, Department of Pharmacy, Sri Lanka.

^6^University of Newcastle, Clinical Toxicology Research Group, Newcastle, New South Wales, Australia

^7^The University of Sydney, Discipline of Clinical Medicine & Addiction Medicine, Faculty of Medicine and Health, The University of Sydney, NSW, Australia

^8^Drug Health Services, Royal Prince Alfred Hospital, Camperdown, NSW, Australia

**^ⱡ^**Shared senior authors

*Correspondence: Nicholas A Buckley, Level 3, 1-3 Ross St (K06), The University of Sydney, Sydney, NSW 2006, [nicholas.buckley@sydney.edu.au](mailto:nicholas.buckley@sydney.edu.au)

*Fathima Shihana, Level 3, 1-3 Ross St (K06), The University of Sydney, Sydney, NSW 2006, [fhan0023@sydney.edu.au](mailto:fhan0023@sydney.edu.au)

Supplementary Table 1: The selected microRNAs for testing in the validation cohort

| **miR** | **Assay ID** | **Source** |
| --- | --- | --- |
| hsa-miR-10a | TM000387 | Discovery-Urine |
| hsa-miR-15b | TM000390 | Discovery-Urine |
| hsa-miR-16 | TM000391 | Discovery-Urine |
| hsa-miR-17 | TM002308 | Discovery-Urine |
| hsa-miR-19a | TM000395 | Discovery-Urine |
| hsa-miR-19b | TM000396 | Discovery-Urine |
| hsa-miR-20a | TM000580 | Discovery-Urine |
| hsa-miR-21 | TM000397 | Discovery-Urine |
| hsa-miR-24 | TM000402 | Discovery-Urine |
| hsa-miR-25 | TM000403 | Discovery-Urine |
| hsa-miR-26a | TM000405 | Discovery-Urine |
| hsa-miR-27a | TM000408 | Literature |
| hsa-miR-29a | TM002112 | Discovery-Urine |
| hsa-miR-29c | TM000587 | Discovery-Urine |
| hsa-miR-30a-3p | TM000416 | Discovery-Urine |
| hsa-miR-30a-5p | TM000417 | Discovery-Urine |
| hsa-miR-30b | TM000602 | Discovery-Urine |
| hsa-miR-30c | TM000419 | Discovery-Urine |
| hsa-miR-30d | TM000420 | Discovery-Urine |
| hsa-miR-30e-3p | TM000422 | Discovery-Urine |
| hsa-miR-92a | TM000431 | Discovery-Urine |
| mmu-miR-93 | TM001090 | Discovery-Urine |
| hsa-miR-106a | TM002169 | Discovery-Urine |
| hsa-miR-106b | TM000442 | Discovery-Urine |
| hsa-miR-122 | TM002245 | Discovery-Urine |
| hsa-miR-125b | TM000449 | Discovery-Urine |
| hsa-miR-126 | TM002228 | Discovery-Urine |
| hsa-miR-130a | TM000454 | Discovery-Urine |
| hsa-miR-142-3p | TM000464 | Literature |
| hsa-miR-146a | TM000468 | Discovery-Urine |
| hsa-miR-148a | TM000470 | Discovery-Urine |
| hsa-miR-155 | TM002623 | Literature |
| hsa-miR-181a | TM000480 | Discovery-Urine |
| hsa-miR-191 | TM002299 | Discovery-Urine |
| hsa-miR-192 | TM000491 | Discovery-Urine |
| hsa-miR-193b | TM002367 | Discovery-Urine |
| hsa-miR-195 | TM000494 | Discovery-Urine |
| hsa-miR-197 | TM000497 | Discovery-Urine |
| hsa-miR-203 | TM000507 | Discovery-Urine |
| hsa-miR-204 | TM000508 | Discovery-Urine |
| hsa-miR-213 | TM000516 | Literature |
| hsa-miR-221 | TM000524 | Discovery-Urine |
| hsa-miR-223 | TM002295 | Discovery-Urine |
| hsa-miR-320 | TM002277 | Discovery-Urine |
| hsa-miR-335 | TM000546 | Discovery-Urine |
| hsa-miR-374 | TM000563 | Discovery-Urine |
| hsa-miR-423-5p | TM002340 | Literature |
| mmu-miR-451 | TM001141 | Discovery-Urine |
| hsa-miR-484 | TM001821 | Discovery-Urine |
| hsa-miR-574-3p | TM002349 | Literature |
| hsa-miR-590-5p | TM001984 | Literature |
| hsa-miR-660 | TM001515 | Discovery-Urine |
| hsa-MIR-1274B | TM002884 | Discovery-Urine |
| ath-mir-159a | | Control |
| ath-miR-172a | | Control |
| ath-mir-394a | | Control |

The microRNAs were selected based on of their significant difference and fold change in the discovery study (Discovery-Urine) or from past literature (Literature). Three microRNA controls (Control) were also included.

Supplementary Table 2: Clinical characteristics of patients and healthy controls included in the microRNA profiling for validation phase (cohort).

| **Validation phase**  **Russell’s viper bite** | HC* | NOAKI | AKIN1 | AKIN2 | AKIN3 |
| --- | --- | --- | --- | --- | --- |
|  | (N=27) | (N=13) | (N=15) | (N=10) | (N=19) |
| Age^a^ | 26 (24-27) | 32 (31-50) | 42 (32-45) | 44 (35-53) | 55 (49-59) |
| Gender % (Female) | 35% | 23% | 20% | 8% | 8% |
| Venom concentration (ng/ml)^a^ | N/A | 8 (0-469) | 23.5 (3-247) | 393 (6-682) | 406 (347-1329) |
| International Normalized Ratio (INR) ^a^ | N/A | 12 (12-12) | 12 (1-12) | 12 (2-12) | 12 (12-12) |
| Time to admission from ingestion (Hour)^a^ | N/A | 1.3 (0.9-2.1) | 1.5 (0.9-2.5) | 1.0 (0.7-1.9) | 1.7 (0.9-2.6) |
| Time to sample collection from ingestion (Hour)^a^ | N/A | 6.1 (5.1-8.0) | 4.1 (2.5-5.9) | 3.8 (3.5-5.5) | 5.1 (2.9-6.7) |
| SCr at the time of sample collection (mg/dl)^a^ | 0.80 (0.71-0.90) | 0.99 (0.83-2.20) | 1.05 (0.82-1.14) | 1.02 (0.70-1.22) | 1.59 (1.06-1.93) |
| 24 hours Peak SCr (mg/dl)^a^ | N/A | 1.10 (0.90-2.50) | 1.16 (1.06-1.28) | 1.35 (1.14-1.47) | 2.60 (2.13-3.41) |
| **Glyphosate** | HC | NOAKI | AKIN1 | AKIN2 | AKIN3 |
|  | (N=27) | (N=17) | (N=19) | (N=8) | (N=7) |
| Age^a^ | 26 (24-27) | 18 (16-28) | 30 (27-37) | 56 (29-66) | 44 (22-56) |
| Gender % (Female) | 35% | 18% | 16% | 0% | 0% |
| Estimated amount ingested (ml)^a^ | N/A | 11 (08-21) | 36 (20-80) | 200 (21-200) | 120 (85-200) |
| Time to admission from ingestion (Hour)^a^ | N/A | 2.5 (2.1-3.7) | 2.1 (0.9-2.9) | 4.2 (2.5-5.5) | 3.7 (2.3-5.4) |
| Time to sample collection from ingestion (Hour)^a^ | N/A | 4.5 (3.4-8.2) | 4.5 (4.0-8.0) | 6.5 (5.0-8.0) | 5.0 (4.0-8.0) |
| SCr at the time of sample collection (mg/dl)^a^ | N/A | 0.88 (0.72-0.98) | 0.94 (0.80-1.14) | 1.53 (1.40-2.92) | 1.14 (0.89-1.90) |
| 24 hours Peak SCr (mg/dl)^a^ | 0.80 (0.71-0.90) | 0.90 (0.81-1.01) | 0.98 (0.94-1.31) | 1.73 (1.53-1.98) | 1.90 (1.14-2.80) |
| Fatal outcome | N/A | 0 | 0 | 0 | 2 |
| **Oxalic acid** | HC | NOAKI | AKIN1 | AKIN2 | AKIN3 |
|  | (N=27) | (N=14) |  | (N=10) | (N=16) |
| Age^a^ | 26 (24-27) | 20 (19-23) |  | 25 (19-28) | 25 (20-28) |
| Gender % (Female) | 35% | 64% |  | 60% | 38% |
| Estimated amount ingested (g)^a^ | N/A | 12.5 (3.1–12.5) |  | 12.5 (8.7-12.5) | 13.4 (12.5-25) |
| Time to admission from ingestion (Hour)^a^ | N/A | 1.4 (1.0-2.0) |  | 2.0 (1.7-3.9) | 3.7 (2.2-4.6) |
| Time to sample collection from ingestion (Hour)^a^ | N/A | 6.6 (4.8-8.0) |  | 6.8 (5.5-8.0) | 6.6 (4.3-8.3) |
| SCr at the time of sample collection (mg/dl)^a^ | 0.80 (0.71-0.90) | 0.72 (0.66-0.78) |  | 0.88 (0.79-0.99) | 1.61 (1.34-1.77) |
| 24 hours Peak SCr (mg/dl)^a^ | N/A | 0.83 (0.73-0.87) |  | 1.35 (0.92-1.91) | 2.58 (1.80-2.90) |
| **Paraquat** | HC | NOAKI | AKIN1 | AKIN2 | AKIN3 |
|  | (N=27) | (N=11) | (N=10) | (N=09) | (N=20) |
| Age^a^ | 26 (24-27) | 24 (23-30) | 23(19-32) | 32 (19-41) | 26 (24-35) |
| Gender % (Female) | 35% | 27% | 20% | 33% | 50% |
| Estimated amount ingested (ml)^a^ | N/A | 15 (10-35) | 20 (10-41) | 20 (20-50) | 20 (20-50) |
| 24 h maximum urine-paraquat levels (ng/ml) | N/A | 16.0(1.0-153.2) | 14.0(1.0-123.6) | 89.5(89.2-298.4) | 387.0(27.1-832.0) |
| Time to admission from ingestion (Hour)^a^ | N/A | 3.5 (2.1-8.7) | 2.5 (2.1-4.0) | 3.0 (2.1-3.9) | 3.3 (2.0-4.8) |
| Time to sample collection from ingestion (Hour)^a^ | N/A | 5.2 (4.9-8.0) | 5.8 (3.1-8.4) | 4.0 (3.5-8.0) | 6 (4.0-8.0) |
| SCr at the time of sample collection (mg/dl)^a^ | 0.80 (0.71-0.90) | 0.82 (0.71-0.92) | 0.81 (0.70-0.92) | 1.20 (0.87-2.19) | 1.09 (0.88-1.48) |
| 24 hours Peak SCr (mg/dl)^a^ | N/A | 0.92 (0.70-0.92) | 0.95 (0.78-1.05) | 1.71 (0.93-2.64) | 1.32 (0.96-1.84) |
| Fatal outcome (N) | N/A | 0 | 0 | 1 | 10 |

^a^ refers to the data expressed as medians and inter quartile ranges (IQRs). HC* represent healthy controls and the same sets of healthy controls used for the comparison in each poisoning.

Supplementary Table 3: Fold change of 43 significantly altered microRNAs in all causes of AKI (AKIN2/3 - NOAKI). Difference values are presented as fold change (2^△Ct^).

| miR IDs | **Russell’s viper bite** | **Paraquat** | **Oxalic acid** | **Glyphosate** | |
| --- | --- | --- | --- | --- | --- |
| miR-30b | 14.2 | 27.3 | 41.6 | 26.4 |  |
| miR-204 | 18.2 | 29.6 | 22.0 | 82.9 |  |
| miR-30a-5p | 58.8 | 19.3 | 49.5 | 14.1 |  |
| miR-191 | 233.7 | 154 | 103.3 | 27.5 |  |
| miR-660 | 62.0 | 20.7 | 7.5 | 9.9 |  |
| miR-30a-3p | 16.3 | 83.0 | 21.0 | 105.5 |  |
| miR-423-5p | 109.7 | 21.8 | 3.0 | 11.9 |  |
| miR-16 | 18.0^a^ |  |  |  |  |
| miR-484 | 79.2^a^ |  |  |  |  |
| miR-181a | 12.1^a^ |  |  |  |  |
| miR-130a | 78.2^a^ |  |  |  |  |
| miR-30e-3p | 8.3^a^ |  |  |  |  |
| miR-15b |  | 11.2^b^ |  |  |  |
| miR-21 |  | 103.3^b^ |  |  |  |
| miR-30c |  | 1362.4^b^ |  |  |  |
| miR-1274b |  | 254.4^b^ |  |  |  |
| miR-320 |  | 49.5^b^ |  |  |  |
| miR-146a |  |  | 18.4^c^ |  |  |
| miR-223 |  |  | 16.6^c^ |  |  |
| miR-20a | 40.2 | 39.7 | 48.0 |  |  |
| miR-192 | 137.0 | 41.5 | 44.3 |  |  |
| miR-10a | 787.6 | 139.1 | 15.7 |  |  |
| miR-203 | 91.2 | 45.4 | 29.6 |  |  |
| miR-335 | 88.3 | 5.9 | 3.8 |  |  |
| miR-92a | 69.5 | 101.2 |  | 25.5 |  |
| miR-195 | 11.3 |  | 2.7 | 21.1 |  |
| miR-24 | 84.9 | 88.1 |  |  |  |
| miR-197 | 506.1 | 164.8 |  |  |  |
| miR-26a | 84.1 | 106.0 |  |  |  |
| miR-29a | 178.0 | 131.9 |  |  |  |
| miR-574-3p | 39.6 | 31.2 |  |  |  |
| miR-19a |  | 8.3 | 3.3 |  |  |
| miR-19b |  | 387.7 | 51.6 |  |  |
| miR-148a |  | 3.4 | 4.3 |  |  |
| miR-221 |  | 9.7 | 10.2 |  |  |
| miR-29c |  | 92.2 | 22.6 |  |  |
| miR-93 |  | 21.3 | 4.8 |  |  |
| miR-451 |  | 5.2 | 5.3 |  |  |
| miR-106a |  | 92.7 | 21.5 |  |  |
| miR-17 |  | 50.6 | 18.5 |  |  |
| miR-142-3p |  | 6.6 | 4.1 |  |  |
| miR-30d | 105.7 |  | 10.3 |  |  |
| miR-193b |  | 184.4 |  | 15.9 |  |

^a, b,^ and ^c^ denote microRNAs specifically differentiated AKI in Russell’s viper bites, paraquat, oxalic acid respectively (Figure 3c).

Supplementary Figure 1: Logistic regression receiver operator characteristics area-under-curve (ROC-AUC) showing the diagnostic performance of the combination seven microRNAs (Table 1) and separately the combination of only the top four microRNAs that distinguished AKI in all poisoning was assessed using normalized Ct between NOAKI(n=57) vs AKIN2/3(n=94).

Supplementary Figure 2: A schematic diagram shows the initial discovery, through validation and selection of four common microRNAs in all four toxic agents (Oxalic Acid, Glyphosate,

Paraquat and Russell’s viper envenoming).
